# Alternative Splicing Regulation of an Alzheimer’s Risk Variant in CLU

**DOI:** 10.3390/ijms21197079

**Published:** 2020-09-25

**Authors:** Seonggyun Han, Kwangsik Nho, Younghee Lee

**Affiliations:** 1 Department of Biomedical Informatics, University of Utah School of Medicine, Salt Lake City, UT 84108, USA; seonggyun.han@utah.edu; 2 Department of Radiology and Imaging Sciences and Indiana Alzheimer Disease Center, Indiana University School of Medicine, Indianapolis, IN 46202, USA; 3 Center for Computational Biology and Bioinformatics, Indiana University School of Medicine, Indianapolis, IN 46202, USA

**Keywords:** *CLU*, Alzheimer’s disease, alternative splicing, sQTLs

## Abstract

Clusterin (*CLU*) is one of the risk genes most associated with late onset Alzheimer’s disease (AD), and several genetic variants in *CLU* are associated with AD risk. However, the functional role of known AD risk genetic variants in *CLU* has been little explored. We investigated the effect of an AD risk variant (rs7982) in the 5th exon of *CLU* on alternative splicing by using an integrative approach of brain-tissue-based RNA-Seq and whole genome sequencing data from Accelerating Medicines Partnership—Alzheimer’s Disease (AMP-AD). RNA-Seq data were generated from three regions in the temporal lobe of the brain—the temporal cortex, superior temporal gyrus, and parahippocampal gyrus. The rs7982 was significantly associated with intron retention (IR) of the 5th exon of *CLU*; as the number of alternative alleles (*G*) increased, the IR rates decreased more significantly in females than in males. Our results suggest a sex-dependent role of rs7982 in AD pathogenesis via splicing regulation.

## 1. Introduction

Alternative splicing (AS) is associated with many neurodegenerative disorders, including AD [1,2,3], and many genetic variants that contribute to diseases affect splicing regulation [4]. Accumulating evidence further suggests that AD susceptibility variants may affect alternative splicing [5].

*CLU* (clusterin) is associated with AD and is known to influence the neurotoxic effects of Aβ deposition [6,7]. *CLU* is largely regulated by AS mechanisms [3,8]. Most importantly, *CLU* may be a factor that contributes to greater AD vulnerability in females, suggesting that risk variants of *CLU* could have sex-specific effects [9]. While gene-level expression represents the sum of all isoform expression, an AS study explores isoform-level expression. Indeed, spliced isoforms can be affected by SNPs located in a gene body (i.e., in exons and introns), with those effects being connected to the binding affinity of splicing factors for the RNA. Thus, rather than overall gene expression, the expression ratio of *CLU* isoforms may provide new additional insights into the functional analysis of associated SNPs. However, the functional effects of known AD risk loci in *CLU* on alternative splicing remain largely unexplored.

One such variant, rs7982, is located within the 5th exon of *CLU*; an alternative splicing event divides this exon into two parts separated by a short intron [6]. Retention of this intron as part of the 5th exon encodes a cleavage site [10]. Therefore, we investigated a sex-specific functional role of rs7982 in AS regulation using brain-tissue-based RNA-sequencing (RNA-Seq) and whole genome sequencing (WGS) data from two independent cohorts.

## 2. Results

### 2.1. Regulatory Function of rs7982 in Splicing

**Figure 1:**
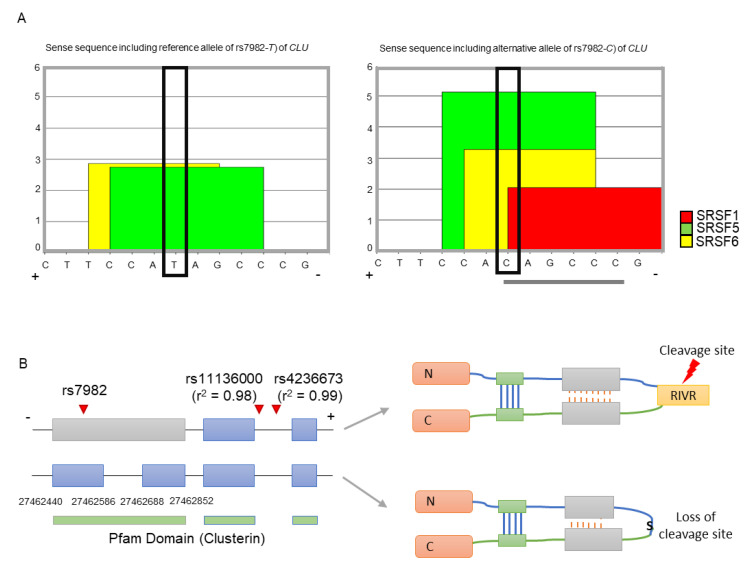
Splicing modulation and functional impact of rs7982. (**A**) The reference allele (T) is involved in two binding motif, SRSF5 and SRSF6 (left figure), while the alternative allele (C) is involved in three motifs, SRSF1, SRSF5, and SRSF6 (right figure). Gray lines underneath indicate each hexameric exonic splicing enhancer (ESE) sequence. The black box indicates allele rs7982 in the binding motif. (**B**) The SNP is located in the 5th exon of CLU. Isoforms retaining the intron (upper transcript) are translated into normally functioning proteins containing the RIVR (Arg-Ile-Val-Arg) sequence, which is part of a key cleavage site; isoforms without the retained intron (lower transcript) produce dysfunctional proteins lacking the cleavage site. The + and – refers a DNA strand as a template. N and C is the N-terminal and C-terminal domain respectively. S indicates serine amino acid.

As *CLU* is a minus-stranded gene, we used the sense sequence of *CLU* (i.e., the reference allele is *T* and alternative allele is *C*) for the splicing regulatory element (SRE) and splicing binding site analyses (see Methods and Materials). We found that the only alternative allele (*C*, major) of rs7982 has exonic splicing enhancer (ESE) hexamers, namely *C*AGCCC (Figure 1A). Furthermore, the *C* allele corresponded to the binding motifs of three splicing factors (SRSF5, SRSF6, and SRSF1), while the *T* allele contributed to a motif for two splicing factors (SRSF5 and SRSF6) (Figure 1A). Furthermore, the binding affinities of SRSF5 and SRSF6 are greater in the alternative allele *C* than the reference allele *T*. This suggests that even though both may form splicing enhancers, the alternative allele may constitute the stronger enhancer. Therefore, rs7982G may promote alternative splicing, specifically division of the 5th exon of *CLU*. Transcripts without the retained intron may be dysfunctional due to loss of a functional cleavage site, RIVR (Arg-Ile-Val-Arg) (Figure 1B).

### 2.2. Association of Intron Retention (IR) with rs7982

**Figure 2:**
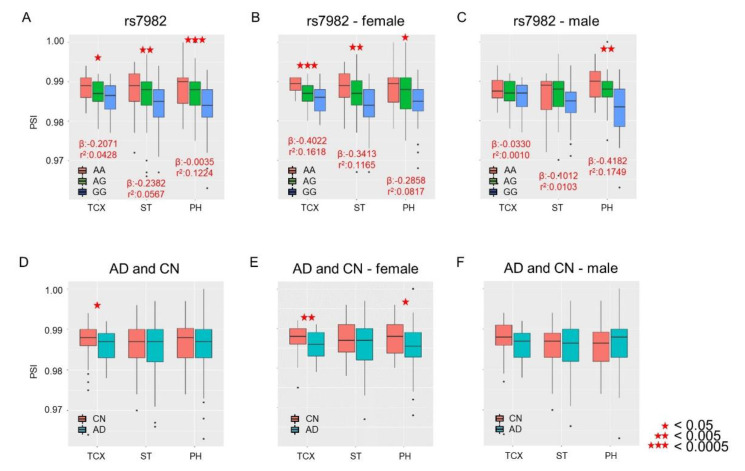
Associations of PSI levels of the 5th exon of *CLU* with rs7982 and AD diagnosis. The *X*-axis represents temporal lobe regions: temporal cortex (TCX), superior temporal gyrus (ST), and Parahippocampal gyrus (PH). The *Y*-axis represents the PSI level, i.e., rate of intron retention (IR). Top: Association of IR with the rs7982 genotype as a continuous variable, with the numbers of alternative alleles (i.e., AA = 0, AG = 1, GG = 2) tallied for all samples (**A**): females only (**B**); males only (**C**). Bottom: Association of IR with AD (cognitively normal older adults (CN) vs. AD) for all samples (**D**): females only (**E**); males only (**F**). Black dots indicate outliers.

We performed splicing quantitative trait loci (sQTLs) analysis to evaluate the associations of percent-spliced-in (PSI) from the 5th exon of *CLU* with the rs7982 genotype and of AD diagnosis with sex as a covariate. Significant associations between PSI and rs7982 were observed in all three brain regions (Figure 2A; temporal cortex (TCX): *p* = 1.02 × 10^−2^; superior temporal gyrus (ST): *p* = 2.34 × 10^−3^; and Parahippocampal gyrus (PH): *p* = 7.03 × 10^−6^). IR rates decreased as the number of *G* alleles increased (Figure 2A). Lower IR levels were also observed in AD in the TCX but not ST and PH regions (Figure 2D; TCX: *p* = 0.041, OR = 0.88; ST: *p* = 0.369, OR = 0.92; and PH: *p* = 0.549, OR = 0.93).

### 2.3. Sex-Dependent Association of IR with rs7982

We examined whether sex is a potential biological factor in rs7982-modulated IR and whether there is an effect of an interaction between rs7982 and sex on IR. Our analysis identified no significant sex-by-rs7982 interaction (TCX: *p* = 0.10; ST: *p* = 0.25; PH: *p* = 0.48). However, in female participants, IR was associated with rs7982 in all three brain regions (TCX: *p* = 1.90 × 10^−4^; ST: *p* = 9.91 × 10^−4^; PH: *p* = 5.74 × 10^−3^, Figure 2B); in male participants, IR was associated with rs7982 only in PH (TCX: *p* = 0.762; ST: *p* = 0.401; PH: *p* = 5.26 × 10^−4^, Figure 2C). In addition, female AD patients had less intron retention in TCX and PH regions (TCX: *p* = 9.10 × 10^−3^; ST: *p* = 0.165; PH: *p* = 0.042, Figure 2E), while male participants demonstrated no difference in IR rates in any of the three temporal lobe regions (TCX: *p* = 0.260; ST: *p* = 0.785; PH: *p* = 0.625, Figure 2F).

## 3. Discussion

Here, we functionally annotated rs7982, a SNP within the 5th exon of *CLU* that is associated with AD and Aβ accumulation. Of the seven exons in *CLU* that encode clusterin domains, only the 5th exon undergoes alternative splicing (i.e., intron retention), conferring a key part of a cleavage site. The full-length mRNA of *CLU* is translated into extracellular secreted CLU (sCLU), while other shorter mRNAs generate intracellular forms (iCLU). A recent study showed that mutations in the 5th exon reduced the sCLU protein and increased iCLU protein [11], and even an absence of the 5th exon tends to be associated with an increased generation of iCLU form [11,12]. However, the function of *CLU* in AD pathology is controversial. Unlike the protective effect associated with a higher extracellular clusterin concentration (sCLU), iCLU is related to cell cytotoxicity, which may be involved in AD progression through interactions with tau and *BIN1* [11,13]; that is, an rs7982G-dependent alternative splicing event may be implicated in AD by affecting the subcellular CLU localization, whose iCLU form is involved in AD progression generated via a partial loss (i.e., less intron retention) of the 5th exon.

We showed a significant sex-dependent association of rs7982 with IR levels in three temporal lobe regions, suggesting that rs7982 may be an important factor underlying AD susceptibility by modulation of IR in the 5th exon of *CLU*. Interestingly, we observed that rs7982 might have a sex-specific effect on IR levels, with IR showing a significantly greater decrease in female AD patients relative to cognitively normal older adults (CN). This difference was significant in two brain regions (TCX and PH; cohort-independent difference), and although not significant in ST, the same tendency was observed. Notably, the alternative splicing event is tissue-dependent, and these events are associated with brain site-specific functions, meaning that splicing may contribute to generating an mRNA isoform that is translated into a protein version specific to the brain site [14,15,16,17]. Therefore, this observation may need to be replicated with a larger sample size and to be validated with further experimental study to investigate why such intron retention is regulated to be brain-region-specific and to discover the underlying mechanism.

The sample size of females is larger than that of males, as shown in Table S1. This difference in the sample size between male and female groups may lead to differing statistical power. However, such a difference in sample sizes between groups may not be a bias in our study, as the sex-dependent effect of rs7982 was much stronger when examining a comparison of the similar sizes between females and males in TCX (Figure 2). Moreover, the sample sizes of both groups in the CN group were similar for each brain region (Table S1). The significant effect of rs7982 in the female CN group was replicated in two similar brain regions, TCX and ST (Figure S1B,E), while the significant effect of rs7982 was not replicated in the CN male group (Figure S1C,F). For AD cases, we observed similar results. In addition, the standardized beta and r^2^ values were higher in females than males in three sample groups, namely CN only (Figure S1), AD only (Figure S2), and CN and AD together (Figure 2). Therefore, we believe that this sex-dependent functional effect of rs7982 may not be due to the difference in sample sizes between sexes. Additionally, we evaluated the potential presence of baseline genotype differences between groups, such as the AD male and AD female groups (Table S2), and none of these comparisons showed a statistical difference (see Materials and Methods and Table S3). *CLU* is considered a factor for increased amyloid dyshomeostasis associated with the earliest female brain transition, providing a mechanistic rationale that the female brain is more vulnerable to AD [9].

Our finding in *CLU* is consistent with the sex-dependency of AD and provides a molecular basis for understanding mechanisms underlying the functional association of risk variants with AD pathogenesis. Notably, rs7982 is in strong linkage disequilibrium with the well-known AD susceptibility SNPs rs11136000 (*r*^2^ = 0.98) [6] and rs4236673 (*r*^2^ = 0.99) [18]. Our results may, therefore, indirectly explain other AD-associated variation. It is well-known that SNPs located within a given AS exon and its flanking introns are likely to be able to affect AS regulation. Thus, AS of the 5th exon could be regulated by rs7982, which is located in that exon. In addition, there is evidence that the haplotypes of these SNPs (GCG and ATC; rs7982, rs11136000, and rs933188, respectively) are related to amyloid beta deposition, thereby influencing the risk of AD in vivo [19]. Thus, there is a possibility that these SNPs have a haplotype-based effect on splicing [20].

There are seven known AD-associated SNPs in *CLU*, according to a previous study [19]. We summarized their minor allele frequencies (Figure S3). As mentioned above, rs7982 is in strong linkage disequilibrium (LD) with rs11136000 and rs4236673, and the minor allele frequencies of those SNPs were greater than those of the other AD-associated SNPs in *CLU*.

Since there is a possibility that our results concerning the splicing rate may be dependent on the analysis method used, we repeated our integrative analysis using another method, MISO, to define and calculate the splicing rate (PSI) based on the Bayesian approach [21]; this method yielded the same results (Figure S4).

We evaluated the ability of rs7982 to enhance a splicing event (less intron retention) within the 5th exon of *CLU* using two different splicing-regulation-related datasets: (1) *cis*-acting regulatory elements, i.e., ESEs; (2) SR splicing factor binding motifs. Both showed consistent indications that the rs7982G allele may increase splicing within the 5th exon of *CLU*. In addition, we analyzed RNA-Seq data generated from three temporal lobe regions in two independent cohorts and identified consistent associations across them. In other words, we integrated RNA-Seq and WGS data to present a priori knowledge- and data-driven evidence that rs7982G (risk allele) tends to elevate AS efficiency (less intron retention). Consistently, decreased IR (loss of functional exon regions) was observed in AD.

## 4. Materials and Methods

### 4.1. RNA-Seq and WGS Analysis

We downloaded RNA-Seq (BAM files) and WGS data (VCF files) from the Synapse database (www.synapse.org). Data were generated from two independent cohorts in the Accelerating Medicines Partnership–Alzheimer’s Disease (AMP-AD) project: the Mayo Clinic Brain Bank [22] and the Mount Sinai Brain Bank (MSBB) [23]. The participant demographics and included brain regions are described in Table S1. RNA-Seq data were generated from three regions in the temporal lobes of CN and AD patients: TCX, ST, and PH. We converted the binary version of the Sequence Alignment/Map (SAM) file (BAM) files into FASTQ files, then mapped the reads to the human reference genome (GRCh37.75) using STAR [24]. We identified AS events and quantified their incidences as the PSI, the fraction of mRNAs including an AS exon, using rMATs [25].

### 4.2. Implication of rs7982 in Splicing Regulation

First, we investigated whether the synonymous variant rs7982 in the 5th exon of *CLU* was located within a splicing regulatory element (SRE, i.e., ESE or exonic splicing silencer (ESS) [26]), a hexameric regulatory sequence in an exonic region. We scanned the surrounding sequence for SREs according to our previously published method [26]. Second, using ESEfinder, we determined whether rs7982 coincided with any splicing factor binding motifs. Because *CLU* is transcribed into minus-stranded RNA, we used the sense sequence (i.e., reverse complimentary sequence of reference DNA sequence) of *CLU* exon 5 (Table S4) in the SRE and splicing binding motif analyses. We compared the surrounding sequence against a database of nucleotide frequency matrices for SR family proteins generated from in vitro tests [27].

### 4.3. Statistical Analysis

We determined allele-specific associations of rs7982 with AS levels (PSI) for each brain region using linear regression with respect to the genotype of rs7982 as a continuous variable, tallying the numbers of alternative alleles (i.e., AA = 0, AG = 1, GG = 2). We then compared PSI values between CN and AD groups by logistic regression, including sex as a covariate. We performed parametric *t*-tests to compare PSI values between CN and AD in each sex group. In addition, we performed ANOVA to evaluate the effect of an interaction between sex and rs7982 on PSI values. We further performed a 2 × 2 Chi-square test on the minor allele frequency to evaluate baseline genotype differences between groups (i.e., AD male vs. AD female, CN male vs. CN female, AD male vs. CN male, and AD female vs. CN female).

## 5. Conclusions

In conclusion, we present new insights into the molecular mechanism of action for rs7982 in *CLU*, which potentially acts as a sex-dependent AD risk factor via alternative splicing.

## Supplementary Materials

Supplementary Materials can be found at https://www.mdpi.com/1422-0067/21/19/7079/s1.

## Abbreviations

TCX	Temporal cortex
ST	Superior temporal gyrus
PH	Parahippocampal gyrus
OR	Odds ratio
AD	Alzheimer’s disease
AMP-AD	Accelerating Medicines Partnership–Alzheimer’s Disease
ESE	Exon skipping enhancer
IR	Intron retention
AS	Alternative splicing
sQTLs	Splicing quantitative trait loci
