# Supplementary material for: Alternative Splicing Regulation of an Alzheimer’s Risk Variant in CLU
Source: Int J Mol Sci. 2020 Sep 25;21(19):7079. doi: 10.3390/ijms21197079 (PMC7582367; doi:10.3390/ijms21197079)
Supplement: Supplementary file 1 [file ijms-21-07079-s001.pdf]

Alternative splicing regulation of an Alzheimer's risk variant in *CLU*

Seonggyun Han [1], Kwangsik Nho [2, 3]*, Younghee Lee [1]*

[1]*Department of Biomedical Informatics, University of Utah School of Medicine, Salt Lake City, Utah, USA;* [2]*Department of Radiology and Imaging Sciences and Indiana Alzheimer Disease Center, Indiana University School of Medicine, Indianapolis, IN, USA;* [3]*Center for Computational Biology and Bioinformatics, Indiana University School of Medicine, Indianapolis, IN, USA*

Supplementary Materials

Table S1. Demographic summary of samples.

	**Mayo**	**MSBB**	
Region	TCX[a]	ST[b]	PH[c]
# of Male/Female	68/81	71/90	65/92
# of samples of AD (Male/Female)	78 (31/47)	101 (40/61)	93 (33/60)
# of samples of CN (Male/Female)	71 (37/34)	60 (31/29)	64 (32/32)
# of Braak stage	-	3.990 (2.00) [0-6]	3.805 (2.056) [0-6]

a. TCX: temporal cortex
b. ST: temporal gyrus
c. PH: parahippocampal gyrus

Table S2. The number of each genotype in both sexes of AD and control.

	TCX[a]						ST[b]						PH[c]					
	Male			Female			Male			Female			Male			Female		
	AA	AG	GG	AA	AG	GG	AA	AG	GG	AA	AG	GG	AA	AG	GG	AA	AG	GG
# of AD + CN	12	29	27	16	38	27	12	27	32	17	44	29	11	24	30	16	45	31
# of AD	6	15	10	8	22	17	7	15	18	11	31	19	6	11	16	9	29	22
# of CN	6	14	17	8	16	10	5	12	14	6	13	10	5	13	14	7	16	9

a. TCX: temporal cortex
b. ST: temporal gyrus
c. PH: parahippocampal gyrus

Table S3. P values of 2 x 2 chi-square test on minor allele frequency of each comparisons across to three brain regions.

	AD male vs. AD female	CN male vs. CN female	AD male vs. CN male	AD female vs. CN female
TCX	0.7610149	0.1164312	0.2832465	0.4219854
ST	0.371105	0.337872	1	1
PH	0.6269994	0.153694	0.9894979	0.338022

a. TCX: temporal cortex
b. ST: temporal gyrus
c. PH: parahippocampal gyrus

Table S4. DNA sequence of CLU exon 5 from 5’ to 3’ (the sense sequence of *CLU*)

CTTGAGGAGTTCCTGAACCAGAGCTCGCCCTTCTACTTCTGGATGAATGGTGACCGCATC<br>GACTCCCTGCTGGAGAACGACCGGCAGCAGACGCACATGCTGGATGTCATGCAGGACCA<br>CTTCAGCCGCGCGTCCAGCATCATAGACGAGCTCTTCCAGGACAGGTTCTTCACCCGGGA<br>GCCCCAGGATACCTACCACTACCTGCCCTTCAGCCTGCCCCACCGGAGGCCTCACTTCTTC<br>TTTCCCAAGTCCCGCATCGTCCGCAGC[1]TTGATGCCCTTCTCTCCGTACGAGCCCCTGAAC<br>TTCCACGCCATGTTCCAGCCCTTCCTTGAGATGATACACGAGGCTCAGCAGGCCATGGAC<br>ATCCACTTCCAT[2]AGCCCGGCCTTCCAGCACCCGCCAACAGAATTCATACGAGG

[1]yellow box indicates the retained intron region of exon 5
[2]position of rs7982

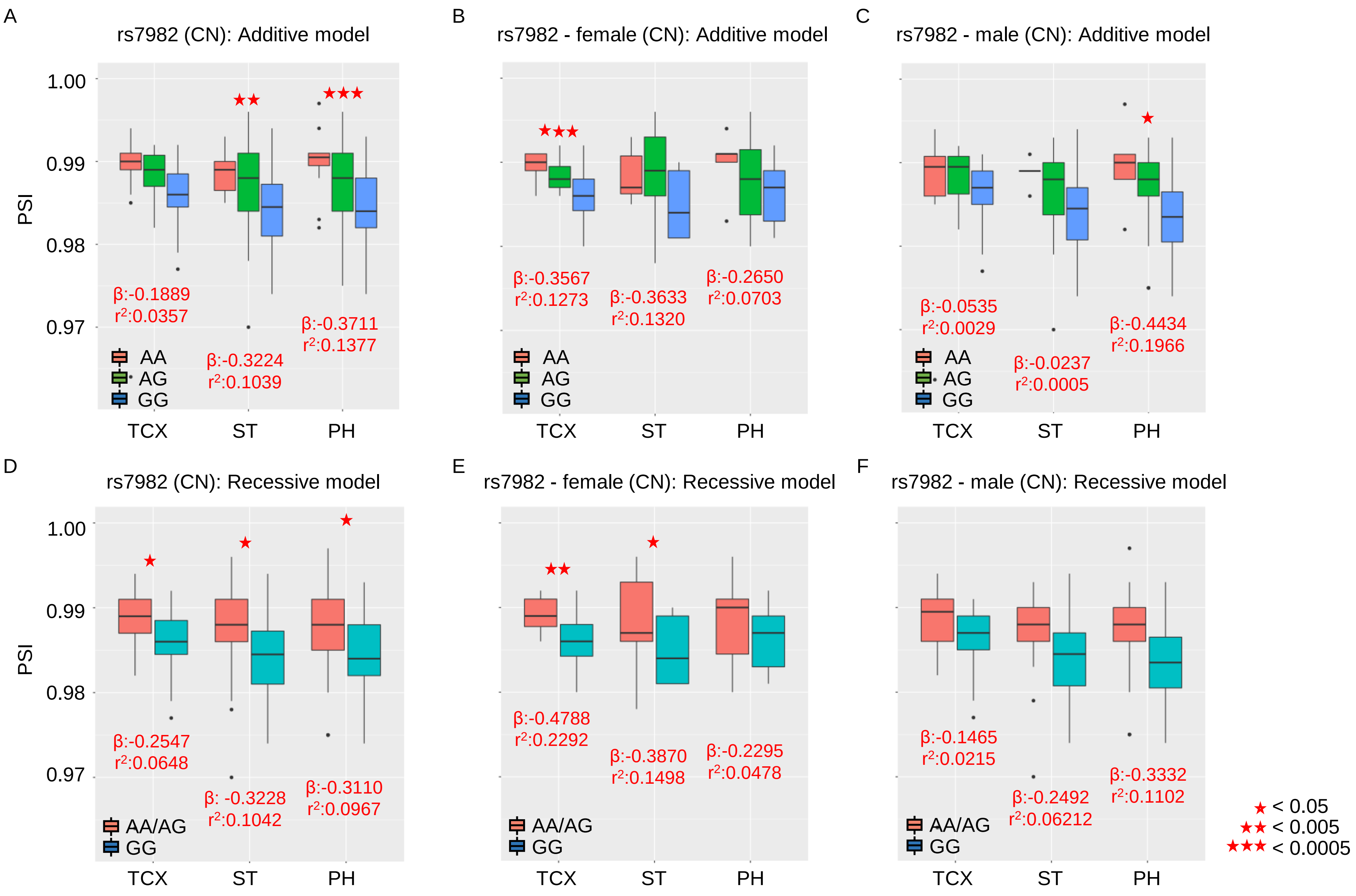

Figure S1. Association of PSI levels of the 5th exon of *CLU* with rs7982 in CN group. X-axis represents temporal lobe region: TCX, ST, and PH. Y-axis represents PSI level, i.e. rate of intron retention (IR). Due to the small number of cases having the minor allele, we present results from both additive and recessive models; the additive model regresses PSI values to the count of minor alleles (i.e. AA=0, AG=1, GG=2) and the recessive model regresses PSI values to the presence or absence of the major allele (i.e. AA and AG=0 and GG=1). Top: Association of IR and rs7982 genotype based on additive model for all samples (A); females only (B); and males only (C). Bottom: Association of IR and rs7982 genotype based on recessive model for all samples (D); females only (E); and males only (F).

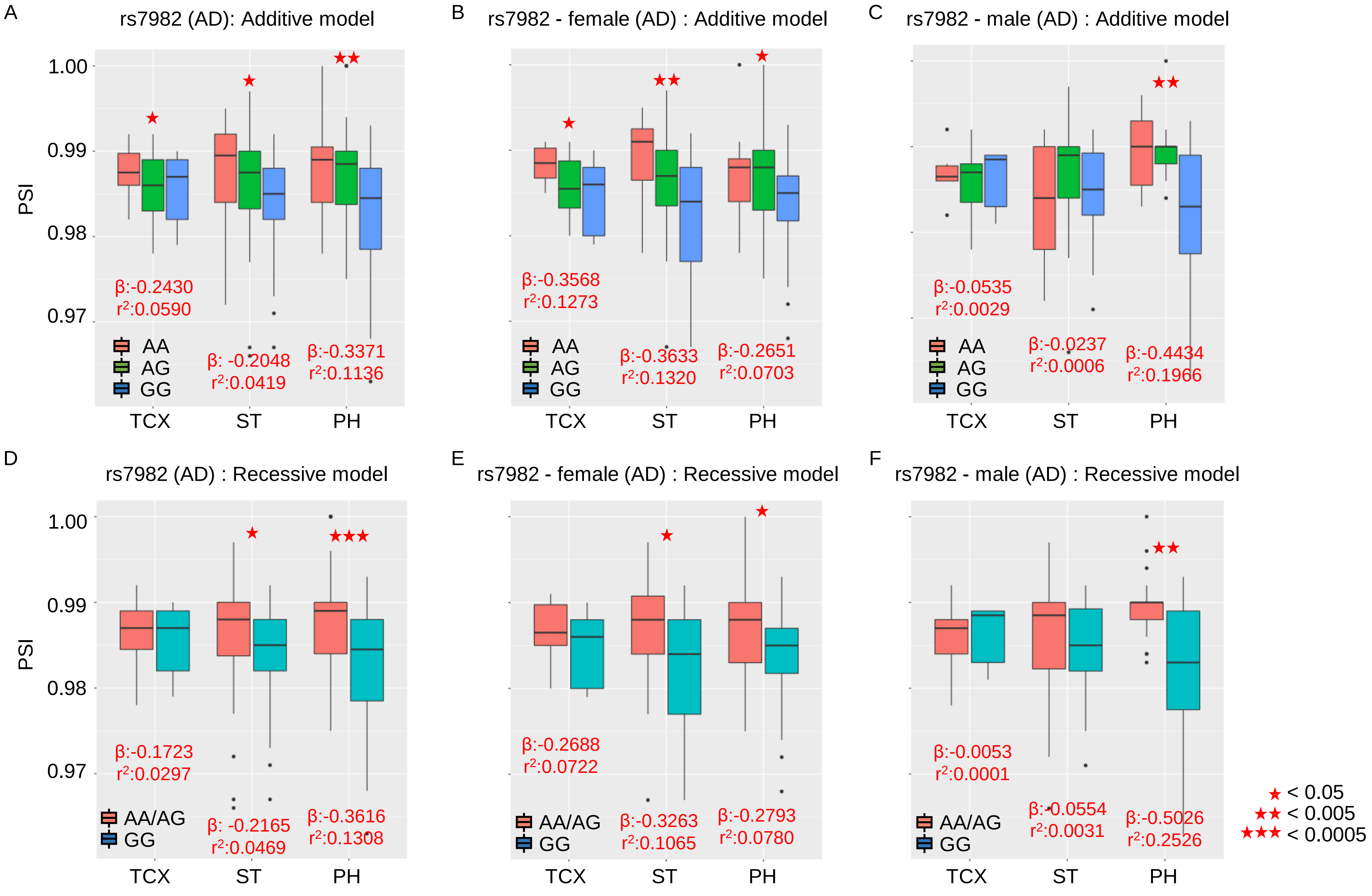

Figure S2. Association of PSI levels of the 5th exon of *CLU* with rs7982 in AD group. X-axis represents temporal lobe region: TCX, ST, and PH. Y-axis represents PSI level, i.e. rate of intron retention (IR). Due to the small number of cases having the minor allele, we present results from both additive and recessive models; the additive model regresses PSI values to the count of minor alleles (i.e. AA=0, AG=1, GG=2) and the recessive model regresses PSI values to the presence or absence of the major allele (i.e. AA and AG=0 and GG=1).
Top: Association of IR and rs7982 genotype based on additive model for all samples (A); females only (B); and males only (C). Bottom: Association of IR and rs7982 genotype based on recessive model for all samples (D); females only (E); and males only (F).

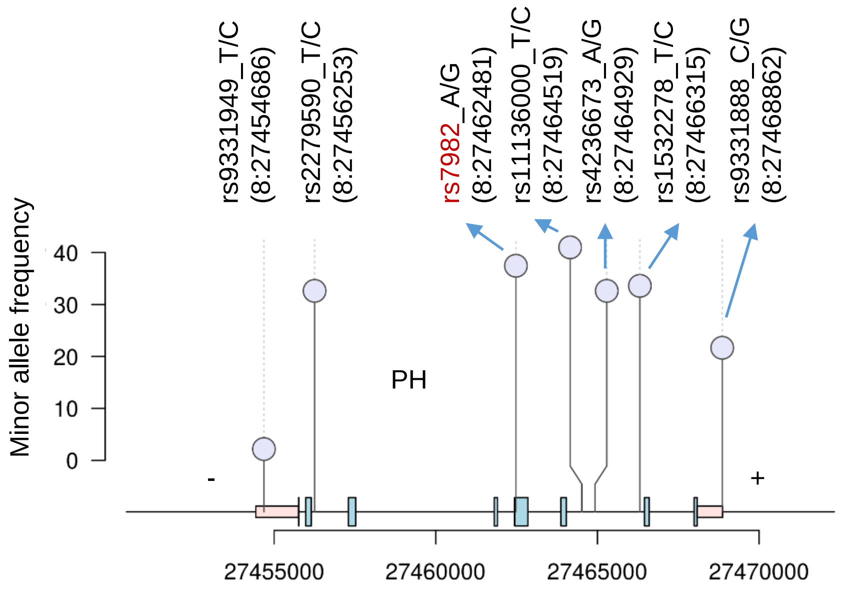

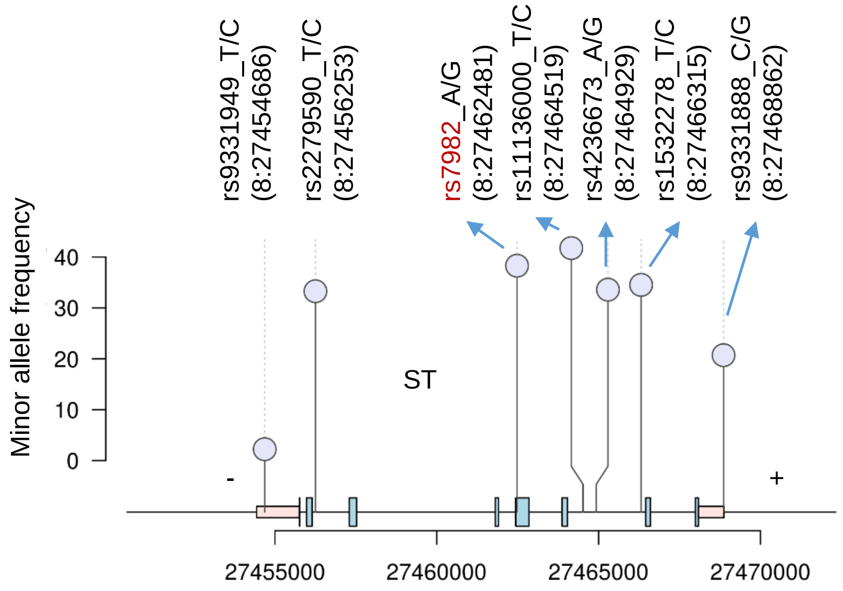

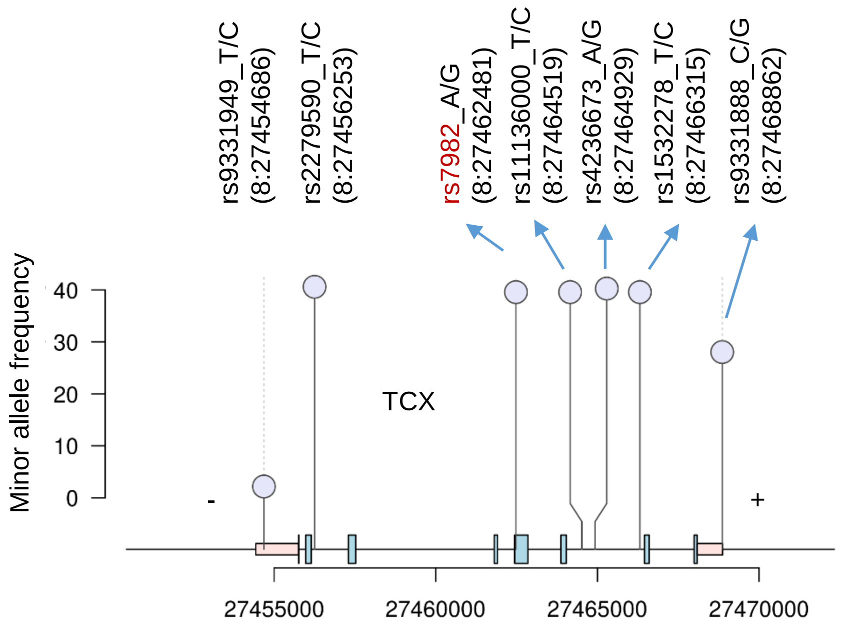

Figure S3. Minor allele frequencies (MAFs) of AD-associated SNPs in CLU by brain region. The x-axis refers to genomic coordinates and the y-axis indicates MAF. Plots were generated by using the trackViewer R package.

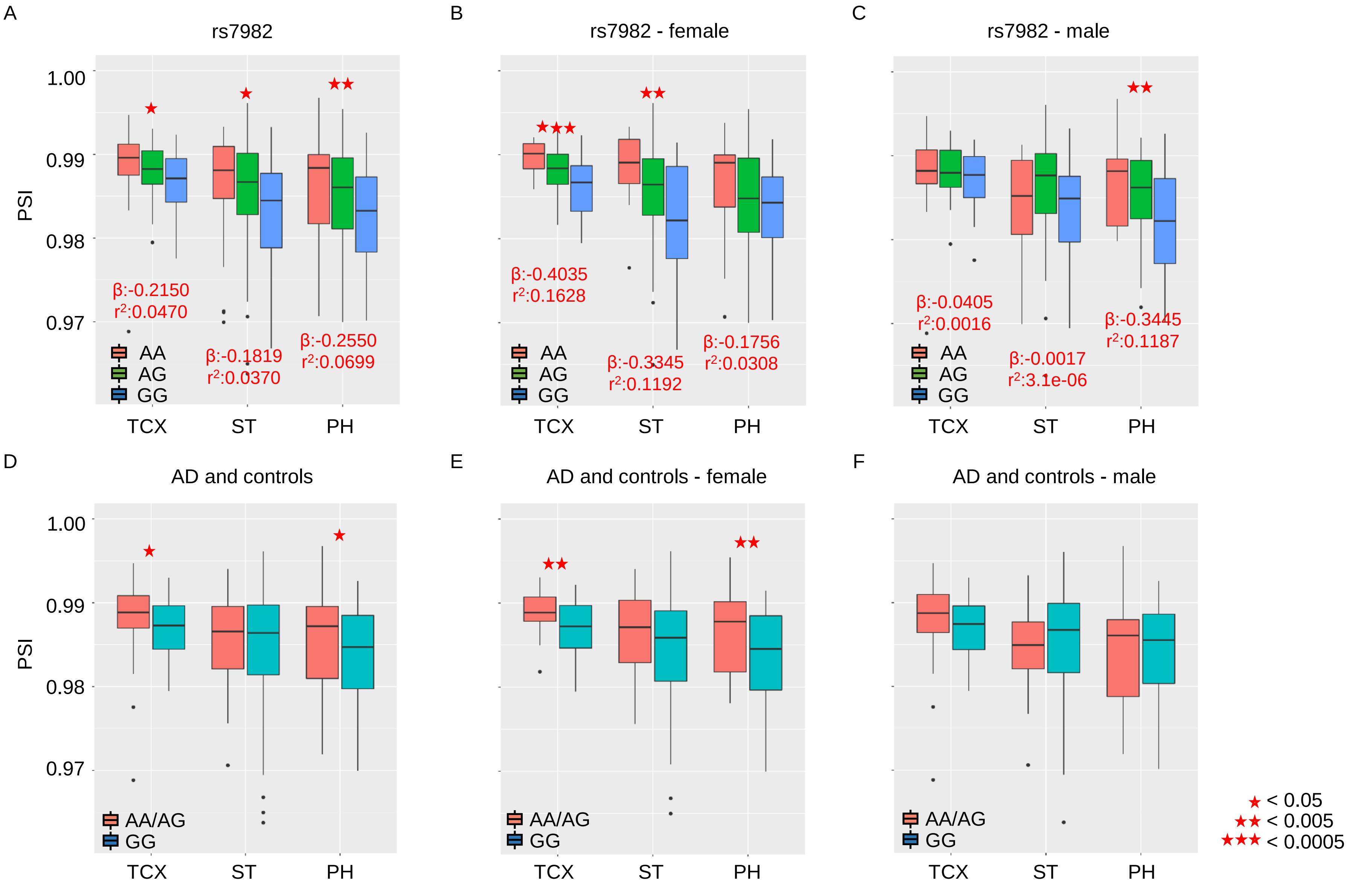

Figure S4. Association of PSI levels of the 5th exon of *CLU* with rs7982 and AD by using the MISO tool. The X-axis represents temporal lobe regions: TCX, ST, and PH. The Y-axis represents PSI level, i.e. rate of intron retention (IR). Top: Association of IR with rs7982 genotype as a continuous variable tallying the number of alternative alleles (i.e. AA=0, AG=1, GG=2) for all samples (A); females only (B); and males only (C). Bottom: Association of IR with AD (CN vs. AD) for all samples (D); females only (E); and males only (F).
